# Prolongation of metallothionein induction combats Aß and α-synuclein toxicity in aged transgenic *Caenorhabditis elegans*

**DOI:** 10.1038/s41598-020-68561-7

**Published:** 2020-07-16

**Authors:** Dagmar Pretsch, Judith Maria Rollinger, Axel Schmid, Miroslav Genov, Teresa Wöhrer, Liselotte Krenn, Mark Moloney, Ameya Kasture, Thomas Hummel, Alexander Pretsch

**Affiliations:** 1 Oxford Antibiotic Group GmbH, Konrad-Lorenz-Straße 24, 3430 Tulln, Austria; 2 Department of Pharmacognosy, University of Vienna, Althanstrasse 14, 1090 Vienna, Austria; 3 Department of Neuroscience and Developmental Biology, University of Vienna, Althanstrasse 14, 1090 Vienna, Austria; 4 Department of Chemistry, University of Oxford, 12 Mansfield Road, Oxford, OX1 3TA UK

**Keywords:** Phenotypic screening, Target identification, Alzheimer's disease

## Abstract

Neurodegenerative disorders (ND) like Alzheimer’s (AD), Parkinson’s (PD), Huntington’s or Prion diseases share similar pathological features. They are all age dependent and are often associated with disruptions in analogous metabolic processes such as protein aggregation and oxidative stress, both of which involve metal ions like copper, manganese and iron. Bush and Tanzi proposed 2008 in the ‘metal hypothesis of Alzheimer’s disease’ that a breakdown in metal homeostasis is the main cause of NDs, and drugs restoring metal homeostasis are promising novel therapeutic strategies. We report here that metallothionein (MT), an endogenous metal detoxifying protein, is increased in young amyloid ß (Aß) expressing *Caenorhabditis elegans,* whereas it is not in wild type strains. Further MT induction collapsed in 8 days old transgenic worms, indicating the age dependency of disease outbreak, and sharing intriguing parallels to diminished MT levels in human brains of AD. A medium throughput screening assay method was established to search for compounds increasing the MT level. Compounds known to induce MT release like progesterone, ZnSO_4_, quercetin, dexamethasone and apomorphine were active in models of AD and PD. Thioflavin T, clioquinol and emodin are promising leads in AD and PD research, whose mode of action has not been fully established yet. In this study, we could show that the reduction of Aß and α-synuclein toxicity in transgenic *C. elegans* models correlated with the prolongation of MT induction time and that knockdown of MT with RNA interference resulted in a loss of bioactivity.

## Introduction

Alzheimer’s-, Parkinson’s-, and Huntington’s or Prion diseases are age-dependent disorders which are characterized by an accumulation of misfolded proteins and neuronal cell death. All current approaches for the treatment of AD provide only temporary symptomatic relief and do not inhibit the underlying disease mechanisms because they have mainly been developed based upon a notion that has been dominating the AD field for the past two decades—**‘**the amyloid cascade hypothesis’^1^. Several investigational drugs that target Aβ have failed to show any correlation between a reduction in amyloid burden and improvement of cognitive functions in large-scale clinical trials^2^. Recently it has been shown that the metalloprotein Aß becomes amyloidogenic upon treatment with stoichiometric amounts of Zn^2+^ and Cu^2+^^3^. In the metal hypothesis^5^, Bush and Tanzi claimed in 2008 that the interaction of metals with the major protein components of NDs like Aß, α-synuclein, huntingtin or prion proteins is the underlying cause of the corresponding diseases^4^. This is not merely due to increased (i.e., toxic) levels of metal exposure, but rather due to a breakdown in the homeostatic mechanisms that compartmentalize and regulate these metals^5^. Chemical agents that restore metal homeostasis could be effective drugs against ND^6^. It has been reported that calcium, copper, iron and manganese increase as a function of age in *C. elegans*, while potassium and phosphorus levels decrease. Further increases in dietary iron accelerated age-related accumulation of insoluble proteins. Metal chelation by CaEDTA attenuated proteotoxicity in an Aß expressing *C. elegans* model and promoted lifespan and health span^7^. The approved use of several medical chelators is limited to genuine situations of metal overexposure (e.g. Wilson’s disease or lead toxicity) or rheumatoid arthritis because the removal of essential metal ions leads to serious adverse effects (e.g. iron-deficiency anaemia), and their use is any case complicated because chelators cannot cross the BBB due to their hydrophilic nature^8^. Bush and Tanzi developed small molecules with more sophisticated properties (e.g. metal-protein attenuation compounds (MPACs)) that serve as metal exchangers and ionophores^5^. The first-generation of MPACs was based on clioquinol (CQ; 5-chloro-7-iodo-8-hydroxyquinoline). CQ was initially shown to dissolve synthetic Aβ-Cu^2+^/Zn^2+^ aggregates and amyloid deposits from post-mortem AD brain^9^. It is the prototype of the novel drug PBT2, which has been effective in Phase 2 clinical trials for AD and HD (10; 11). Both compounds translocate Cu^2+^ and Zn^2+^ into the cell thereby initiating neuroprotective signalling cascades like PI3K and upregulation of metalloproteases. Moreover neurite extension is promoted and dendritic spine density is increased. The mechanism of action prevents the effects of breakdown of metal homeostasis but also rectifies the misbalance^6^. A prominent response pathway involved in the chelation of metal ions involves MTs^12^. MTs are a heterogeneous superfamily of endogenous multipurpose proteins that participate in the transport, homeostasis, and detoxification of heavy metals^13^. While there is considerable variation in MTs within the animal kingdom, all MTs share similarities of being cysteine-rich, and showing a regulatory response to essential and non-essential metal exposure. The primary mode of action for MTs is formation of metal-thiolate bonds and subsequent removal of the metal from the cytoplasm^14^. In AD, rising metal concentration leads to reactions with Aß to form oligomers and aggregates. Metallothionein-III (MT3) released into the cleft by neighbouring astrocytes has the potential to ameliorate this adverse interaction, but this release is decreased in AD^5^. The metal-exchange process between Zn7MT-III and Aβ1-40 Cu^2+^ has recently been elucidated^15^. Many experimental data demonstrated that MTs have a close relation with neuroprotection and neurological diseases in mammals^16–19^. MT-III was shown to be markedly diminished in brains in AD, amyotrophic lateral sclerosis (ALS), PD, prion disease, brain trauma, brain ischemia, and psychiatric diseases^16^. The down-regulation of MT-III in patients as well as in a transgenic mouse model of AD has been proposed to alter copper homeostasis in the brain and then lead to extracellular amyloid pathology^20,21^. Double transgenic mouse models overexpressing MT-III and human SOD1 (modelling amyotrophic lateral sclerosis) exhibited normal levels of copper ions in spinal cords and showed prolonged survival with significant suppression of motor neuron death^22^. Effects of MT-III expression on ALS mice were also explored by using a retrograde viral delivery system^23^. Even when injection of the adenovirus encoding MT-III gene started at the mean age of disease onset in ALS mice (~ 20 weeks), MT-III expression was found to prevent further loss of motor neurons and prolonged lifespan. The importance of MT in maintaining metal homeostasis was further demonstrated in studies involving exposure to heavy metals in MT-1/2 knock out mice, which led to metal toxicity. In contrast, MT-1/2 overexpressing mice were relatively well protected from heavy metal toxicity^24^. In a study of Xu et al., the role of Zn_7_MT3 to protect against AD was investigated by treating APP/PS1 mice with sustained drug release of Zn_7_MT3 directly to the central nervous system. The results demonstrated that Zn_7_MT3 can significantly ameliorate cognitive deficits, improve the morphology and function of hippocampus, regulate metal homeostasis, abolish Aß plaque load, and reduce oxidative stress and neuronal cell apoptosis in APP/PS1 transgenic mice. Therefore investigators assumed that Zn_7_MT3 has potential for applications in AD therapy^25^. Many experiments showed that some agents (e.g. apomorphin, propofol) exert neuron protective effects mainly via up-regulation of MTs^16,26^. Treatment of SHSY-5Y cells with dexamethasone reduced Cu-dependent α-synuclein aggregates significantly by MT induction^27^. Miyazaki and colleagues showed that the expression of MT-III and its mRNA was up-regulated in the healthy aged rat brain. Lipopolysaccharide (LPS) treatment induced expression of MTIII and its mRNA only in young but not in aged rat brain regions. These results suggested that the reduced inducibility of brain MT-III against oxidative stress with aging is related to vulnerability and neurodegeneration of aged brain tissue^28^. There is strong evidence that activation of failed MT induction in age-dependent ND like AD or PD is a promising novel therapeutic target^29,30^. To find promising drug candidates against NDs based on the metal hypothesis many variables must be considered to conduct a single definitive assay: these include the complex interplay between metals, the magnitude of metal: protein interactions and the non-linear change in metals during aging and during the course of disease^6^. These factors are best explored by using a whole animal screening model.

**Figure 1:**
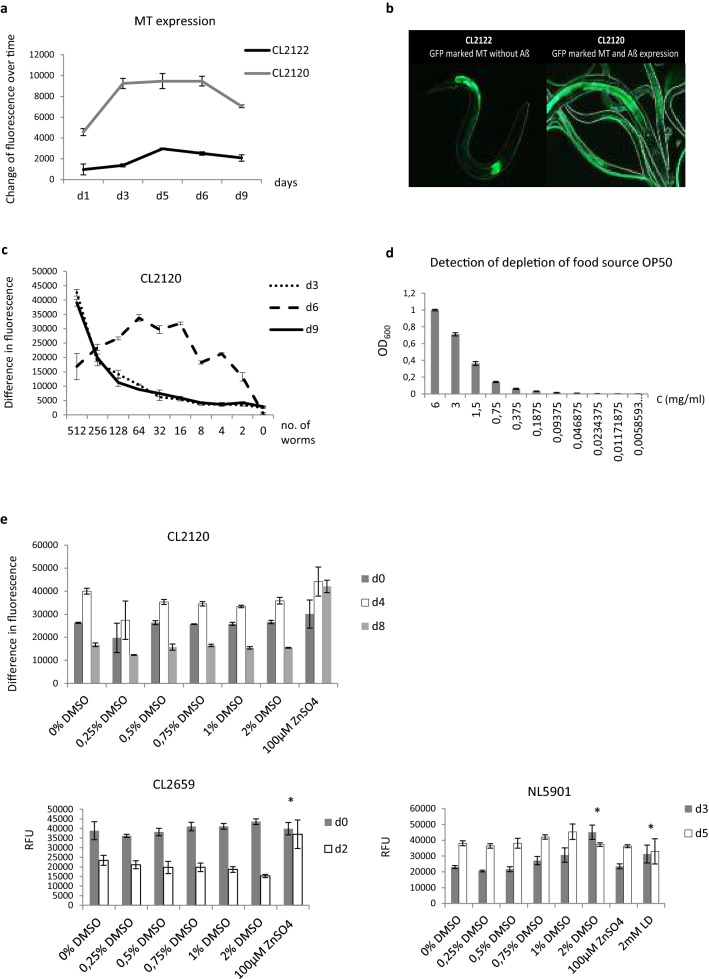
Preliminary tests for a MT medium throughput screening assay based on transgenic *C. elegans*. (**a**) Fluorescence of MT::gfp in strains CL2122 and CL2120 was measured by a fluorescence multiwell plate reader at em/ex 450/535. (**b**) Fluorescence in strains CL2120 and CL2122 was detected at day 6 by fluorescent microscope (magnification ×20). (**c**) Serial dilution (1:1) of CL2120 L4 larvae in a 96 well plate starting with 512 worms/well in triplicates. Fluorescence of reporter was measured after 3, 6 and 9 days. (**d**) Serial dilution (1:1) of *Escherichia coli* strain OP50 in a 96 well plate starting with a concentration of 6 mg/ml. Absorbance was detected by a multiwellplate reader at 600 nm. OD_600_ = 0,8 at a concentration of 5 mg/ml. (**e**) Influence of different DMSO concentrations on fluorescence of GFP reporter in strains CL2120, CL2659 and NL5901. *p ≤ 0.05; **p ≤ 0.005.

**Figure 2:**
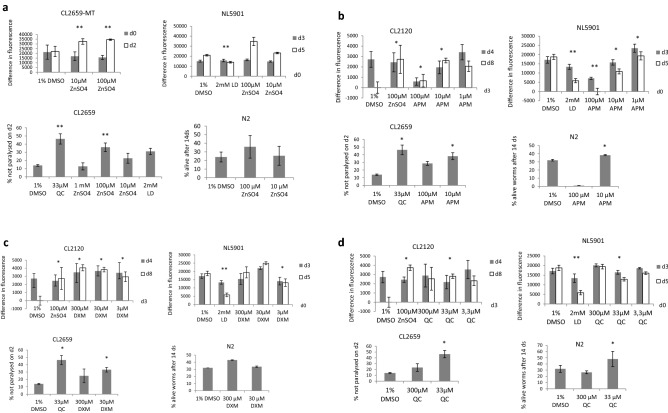
Influence of different concentrations of MT inducing test compounds on strains CL2120, CL2659, NL5901 and N2 was measured by fluorescence multiwell plate reader at em/ex 450/535. Each concentration was tested in triplicates. Change in fluorescence of test compounds was compared to vehicle control 1% DMSO in all assays. (**a**) ZnSO_4_ (**b**) apomorphin (APM) (**c**) dexamethasone (DXM) (**d**) quercetin. Error bars show s.d. *p ≤ 0.05; **p ≤ 0.005; *QC* quercetin, *LD* levodopa, *d* day.

**Figure 3:**
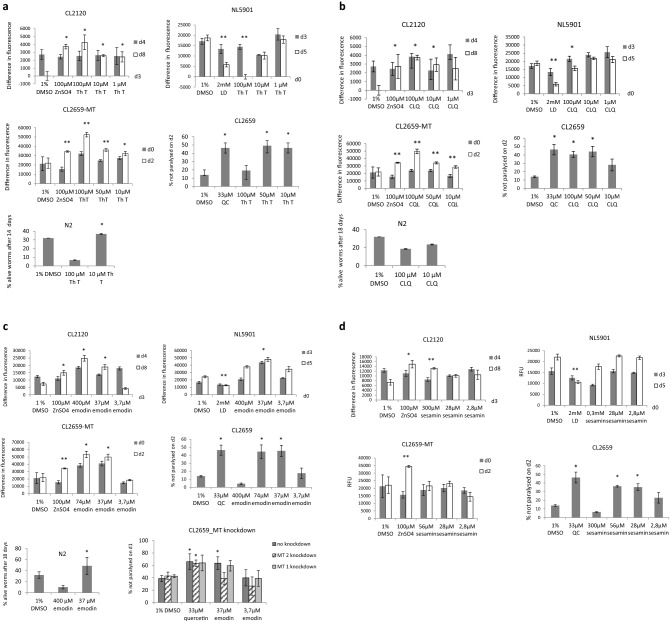
Influence of different concentrations of neuroprotective test compounds on strains CL2120, CL2659, NL5901 and N2 was measured by fluorescence multiwell plate reader at em/ex 450/535. Each concentration was tested in triplicates. Change in fluorescence of test compounds was compared to vehicle control 1% DMSO in all assays. (**a**) thioflavin T (Th T) (**b**) clioquinol (CQL) (**c**) emodin (**d**) sesamin. Error bars show s.d. *p ≤ 0.05; **p ≤ 0.005; *QC* quercetin, *LD* levodopa, *d* day.

The availability of transgenic mouse models is a major step forward in research for NDs, although the associated costs and ethical concerns are a clear drawback^31^. To bridge the gap between in vitro high throughput screening methods and the validation of compounds in mammalian models we used transgenic *C. elegans* models to answer the following question: Can we identify compounds against NDs, which exert their effect via prolongation of MT induction? Therefore, we implemented a robust *C. elegans* medium-throughput assay to monitor MT content during ageing and to investigate the influence of compounds on it. The fully sequenced genome of the nematode *C. elegans* contains two MTs: CeMT-1 is constitutively active in the pharyngeal bulb and CeMT-2 is mainly induced in intestinal cells^12,32^*. C. elegans* is widely used in studies of metal homeostasis, aging and NDs^7,33^. Results from age-related analyses of the metallome indicated that aging of *C. elegans* is associated with the accumulation of iron, copper and manganese. Supplementation with metals can affect worm physiology in different ways. Depending on the concentration, they are able to lengthen or shorten the lifespan and to decrease or increase the pathology of Aß transgenic worms. Iron supplementation enhanced toxicity in both Aß and PolyQ-associated models of protein aggregation^7^. Using transgenic *C. elegans* models of AD and PD we screened several compounds, which have been previously reported to be endowed with ND protective properties. In our experiments in the Aß expressing strain CL2120, where the content of GFP tagged MT can be visualized, the level of MT was increased until day 6 and is followed by a breakdown at day 8. In the healthy control strain CL2122, MT was only slightly induced (Fig. 1). This led to the question of whether the accumulation of metals and the decrease of MT content with ageing might be responsible for the outbreak of AD or PD. To test this, we established a *C. elegans* based medium-throughput screening assay to search for compounds able to prolong MT release in ageing worms. ZnSO_4_, quercetin, apomorphin and dexamethasone induced MT, decreased proteotoxicity of Aß and α-synuclein in strains CL2659 and NL5901 and prolonged lifespan in the wild type strain N2 (Fig. 2). Compounds for novel neurotherapeutics against NDs, such as clioquinol (CQL) thioflavin T (Th T) and emodin decreased proteotoxicity by MT induction and all but clioquinol prolonged lifespan in the wild type strain N2 (Fig. 3). Sesamin was not able to induce MT (Table 1). Knockdown of MT even resulted in a loss of function of the bioactivity of emodin in the AD assay with strain CL2659 (Fig. 3, Table 2).

**Tabel 1:** p-values of the Parkinson-, of the metallothionein-, of the paralysis- and the lifespan assay using student’s t-test.

Compound	Strain				
	CL2120	CL2659	NL5901	CL2659-MT	N2
1% DMSO	1	1	1	1	1
100 µM ZnSO_4_	0.013	0.015	0.778	0.002	0.738
10 µM ZnSO_4_	n.t	0.124	n.t	0.011	0.017
2 mM levodopa	0.773	0.012	0.001	0.531	n.t
100 µM clioquinol	0.038	0.018	0.037	0.001	0.085
50 µM clioquinol	n.t	0.018	n.t	0.001	n.t
10 µM clioquinol	0.018	0.010	0.013	0.002	0.011
1 µM clioquinol	0.245	n.t	0.049		n.t
100 µM thioflavin T	0.011	0.018	0.000	0.000	0.003
50 µM thioflavin T	n.t	0.018	n.t	0.001	n.t
10 µM thioflavin T	0.035	0.010	0.201	0.036	0.009
1 µM thioflavin T	0.045	n.t	0.063	n.t	n.t
100 µM apomorphin	0.044	0.005	0.006	n.t	0.085
10 µM apomorphine	0.013	0.009	0.025	n.t	0.009
1 µM apomorphine	0.163	n.t	0.018	n.t	n.t
300 µM dexamethason	0.030	0.177	0.328	n.t	0.662
30 µM dexamethason	0.035	0.005	0.584	n.t	0.317
3 µM dexamethason	0.056	n.t	0.183	n.t	n.t
300 µM quercetin	0.067	0.128	0.149	n.t	0.051
33 µM quercetin	0.018	0.011	0.007	0.011	0.076
3.3 µM quercetin	0.136	n.t	0.010	n.t	n.t
0.3 µM sesamin	0.005	0.002	0.052	n.t	n.t
56 µM sesamin	n.t	n.t	n.t	0.342	n.t
28 µM sesamin	0.055	0.008	0.109	0.155	n.t
2.8 µM sesamin	0.965	0.123	0.339	0.138	n.t
400 µM emodin	0.008	0.0.003	0.0.014	n.t	0.087
74 µM emodin	n.t	0.023	n.t	0.011	n.t
37 µM emodin	0.018	0.015	0.018	0.040	0.020
3.7 µM emodin	0.002	0.432	0.187	0.014	n.t

*n.t* Not tested.

**Table 2:** p-values of the paralysis assay with RNA interference using student’s t-test.

p-values/RNA interference with CL2659			
	No knock down	MT 2 knock down	MT 1 knock down
1% DMSO	1	1	1
33 µM quercetin	0.05	0.01	0.10
37 µM emodin	0.03	0.59	0.07
3.7 µM emodin	0.90	0.19	0.68

## Results and discussion

### Breakdown of metallothionein induction in aged Aß expressing worms

In 2002 Mijazaki et al.^28^ showed that the expression of MT and its mRNA was up-regulated in the healthy aged rat brain, whereas treatment with lipopolysaccharide (LPS)-induced expression of MT only in young but not in aged rat brain regions. Based on this study, we monitored MT expression in transgenic worms with (CL2120) and without (CL2122) Aß expression. We could show that MT in healthy organisms slightly increases with age whereas in Aß expressing worms an intense induction in the young adults was followed by a breakdown during ageing (Fig. 1a–c) accompanied by an accumulation of iron, copper and manganese^7^. Therefore, we hypothesized that prolonging the time span of MT release might be a promising therapeutic target in NDs.

### Establishment of a novel medium throughput screening assay method to search for compounds that prolong time of MT induction in aged transgenic *C. elegans* model of AD

To screen compounds for their ability to prolong MT induction we established a robust medium throughput screening assay based on *C. elegans* strain CL2120. CL2120 MT is GFP tagged and can be detected by fluorescence in a multiwell plate reader at em/ex 450–535 nm. By a serial dilution step in a multiwell plate, we determined the optimal worm density for a strong signal to be between 30 and 50 worms per well (Fig. 1c). The first measurement (day 0) was performed when larvae in the 4th stage were transferred from the petri dish to the 96 well plate containing media with compounds. The second time point indicated the highest MT level and was between d4 and d7. The last time point indicated the breakdown of MT induction between d7 and d9 (Fig. 1a, c). To guarantee optimal feeding of the worms throughout the whole experiment, we measured the optical density at 600 nm (OD_600_) for the determination of the bacterial clearance. In this way, the change in optical density of bacteria over time was quantified and the necessary amount of *E. coli* OP50 was added based on the absorbance value^34^. The optimal concentration of the *E. coli* food source was determined to be 5 mg/ml with an OD600 of 0.9 (Fig. 1d). A higher concentration might be toxic, whereas less food induces caloric restriction which is known to interfere with several pathways^34^. To enable a broad range of chemically diverse test compounds to be dissolved for assaying, we have chosen DMSO for stock solutions to be diluted for the final test concentration. Accordingly, we screened several test concentrations of DMSO starting with 0, 1–2% in the MT assay and in assays of Aß and α-synuclein toxicity (Fig. 1e). Only a little effect was observed. When assaying α-synuclein toxicity with strain NL5901 the concentration of 2% DMSO turned out to be toxic. Therefore, all test compounds were dissolved in 1% DMSO.

### Compounds known to induce MT protected worms against Aß- and α-synuclein toxicity and prolonged lifespan

For the assay evaluation procedure, we hypothesized that the efficacy of compounds in prolonging the time of MT induction in strains CL2120 or CL2659 will correlate with a reduction in Aß and α-synuclein toxicity burden.

MTs are mainly induced by heavy metals like Zn, Cu, Cd, Hg and others. Constantinidis and Burnet hypothesized that supplementation with zinc could prevent or delay the onset of dementia^35,36^. Many human trials were undertaken e.g. Constantinidis reported about improved memory, understanding, communication and social interaction of AD patients aged between 56 and 86 years when Zn was administered^11^. In our experiment with the transgenic strain CL2659 the supplementation of growth medium with ZnSO_4_ was able to significantly increase the MT level and even prolong timespan where MT is increased in Aß transgenic worms (Fig. 2a: CL2659-MT). Simultaneously we could show that lower concentrations (< 100 µM) decreased whereas higher concentrations (> 100 µM) increased the pathology of Aß (Fig. 2a: CL2659) and α-synuclein (Fig. 2a: NL5901) transgenic worms. 10 µM further prolonged the lifespan of wild type worms N2 (Fig. 2a: N2) Exposure to 100 µM and 200 µM ZnSO_4_ caused cell death in cultured cortical neurons too^37^. Pre-treatment of cortical neurons with 20 µM apomorphin (APM), a dopamine receptor agonist, rescued them from Zn^2+^ toxicity in a dose- and time-dependent manner^37^. Apomorphine is used in clinics for the therapy of PD. It has pleiotropic biological functions because it is antioxidative and upregulates NGF synthesis in cultured mouse astrocytes^38^. Another study reported that apomorphine stimulates degradation of intracellular Aß in a mouse model of AD^39^. The compound also exerts protective effects on neurons mainly via up-regulation of MT^16^. 10 µM apomorphin increased MT in our assay with strain CL2120 too and protected worms from proteotoxicity in both Aß (Fig. 2b: CL2659) and α-synuclein (Fig. 2b: NL5901) assay. Furthermore, the same concentration even prolonged the lifespan in the wild type strain (Fig. 2b: N2).

Glucocorticoids like progesterone are able to induce MT expression^40^. Pre-treatment with the synthetic glucocorticoid analogue dexamethasone (DXM) suppressed the formation of α-synuclein cytoplasmic aggregates in neuroblastoma cells after incubation with copper^27^. In our experiments, 30 µM and 3 µM DXM prolonged MT induction (Fig. 2c: CL2120), reduced proteotoxic burden in strains CL2659 (Fig. 2c: CL2659) and NL5901 (Fig. 2c: NL5901) and 300 µM and 30 µM prolonged the lifespan in the wild type strain N2 (Fig. 2c: N2).

The flavonoid quercetin (QC) was able to induce MT in hepatoma cells and protected them against oxidative stress at a concentration of 10 µM^41^. In *C. elegans* quercetin prolonged the mean lifespan by 15% by increasing stress resistance^42^. Further studies showed that quercetin ameliorated Alzheimer's disease pathology and protected cognitive and emotional function in aged triple transgenic Alzheimer's disease model mice. Extracellular β-amyloidosis, tauopathy, astrogliosis and microgliosis in the hippocampus and the amygdala have been decreased after treatment with quercetin^43^. QC at 33 µM prolonged timespan in which MT content was elevated (Fig. 2d: CL2120) and reduced proteotoxicity in our assays with strains CL2659 (Fig. 2d: CL2659) and NL5901 (Fig. 2d: NL5901). Further the same concentration significantly prolonged lifespan in the wild type strain N2 (Fig. 2d: N2). However, bioactivity of QC in the paralysis assay with CL2659 has not been lost after knockdown of MT with RNA interference (Fig. 3c: CL2659: MT knockdown). One explanation for this could be that QC mediated lifespan extension in *C. elegans* is modulated by age-1, daf-2, sek-1 and unc-43^44^. There has been shown that daf-2 and age-1 are not only responsible for longevity of *C. elegans* but further provide heavy metal resistance^45^. Therefore, we assume that MT induction is not the solely mode of action of QC in AD and PD transgenic nematodes.

### Novel neuroactive lead compounds decreased proteotoxicity of Aß and α-synuclein by prolonging of MT induction in *C. elegans*

After assay evaluation, we investigated if the mode of action of thioflavin T (Th T), clioquinol (CQL), sesamin and emodin against Aß toxicity is based on prolonging the time of MT release. Alavez and colleagues showed that the amyloid binding compound Th T was able to maintain protein homeostasis during aging and extended lifespan and suppressed human Aß associated toxicity in *C. elegans* models depending on the protein homeostasis network regulator heat shock factor 1 (HSF-1), the stress resistance and longevity transcription factor SKN-1, molecular chaperones, autophagy and proteosomal functions^46^. Further treatment with Th T prevented Aß fibrillation in double transgenic AD mice^47^. In our study, Th T prolonged MT induction in both strains CL2120 (Fig. 3a: CL2120) and CL2659 (Fig. 3a: CL2659-MT) at concentrations between 1 µM or 10 µM and 100 µM. We could show for the first time that in the Parkinson assay 100 µM Th T was able to reduce α-synuclein (Fig. 3a: NL5901). In the paralysis assay performed with strain CL2659 (Fig. 3a: CL2659), 50 and 10 µM Th T prolonged the time until the paralysis phenotype indicating a reduction in Aß toxicity. Interestingly 10 µM Th T prolonged whereas 100 µM shortened the lifespan in the wild type strain N2 (Fig. 3a: N2).

**Figure 4:**
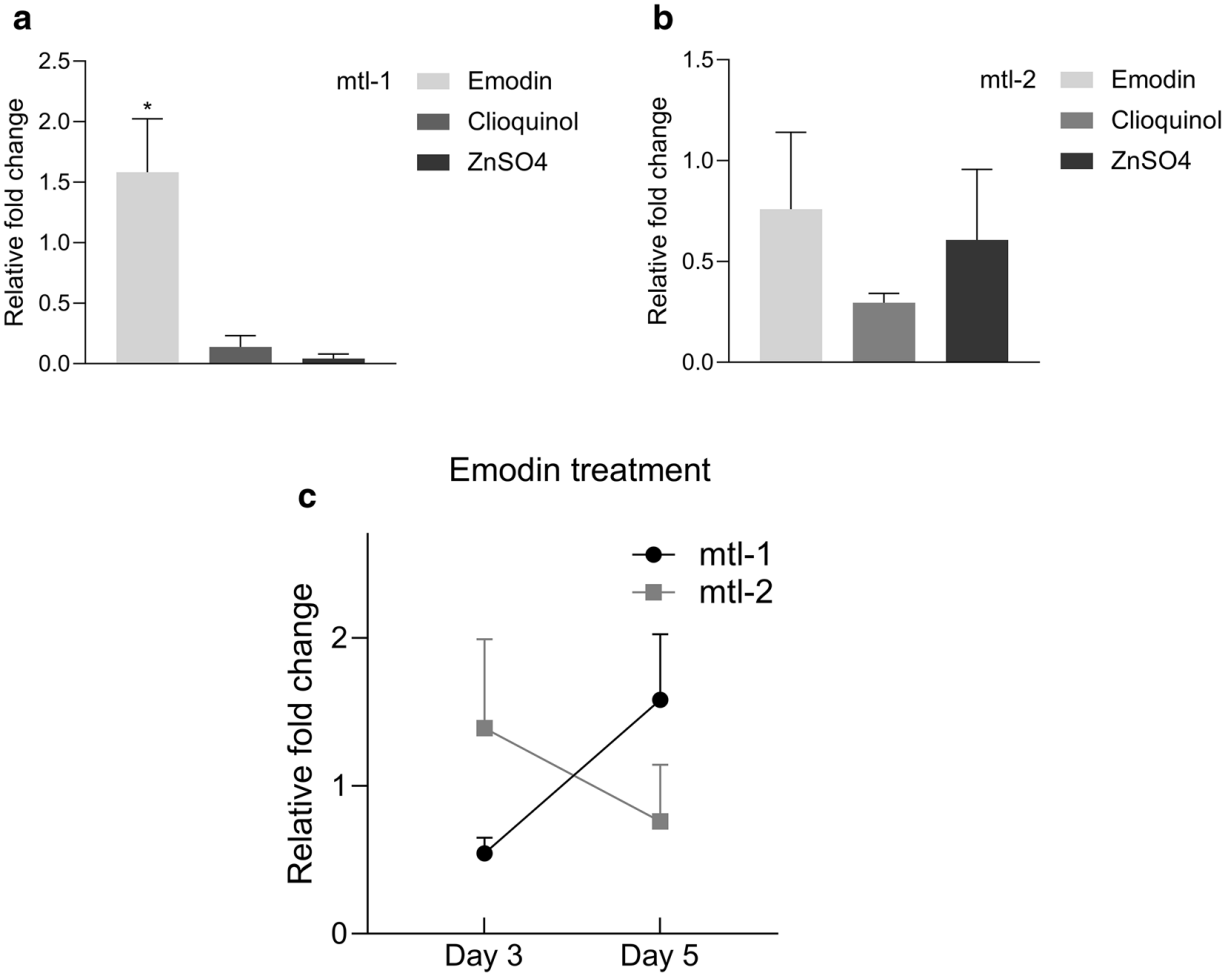
Relative expression of mt-1 (**a**) and mt-2 (**b**) following treatment with test compounds. 150 worms of CL2120 strain were treated with 40 µM emodin, 100 µg/ml clioquinol and 100 µM ZnSO_4_. Relative fold change was measured using comparative ΔΔCT method. Gene expression data was normalized to rps-18. (**c**) 150 worms of CL2120 strain were treated with 40 µM emodin for a period of 3 and 5 days and quantitative RT PCR was performed to measure relative mt-1 and mt-2 expression. One-way ANOVA followed by Tukey's multiple comparisons test was used to measure statistical significance (*p ≤ 0.01). All experiments were performed in triplicates. Data represents means ± SEM.

CQL is the prototype of the novel drug PBT2, which is effective in phase 2 clinical trials for AD and HD^48^. CQL has been shown to be neuroprotective, by decreasing brain aggregate load and restored reduced insulin levels in R6/2 HD mice^49^. A double-blind phase 2 clinical trial demonstrated the efficacy of clioquinol treatment in producing effects on plasma Aβ and zinc ion (Zn^2+^) levels. The drug was well tolerated and inhibited cognitive decline in patients who, untreated, otherwise experienced deterioration^50^. PBT2 delayed the onset of paralysis in a *C. el*egans model of PolyQ overexpression^51^. In our study 10–100 µM CQL induced MT release in strains CL2120 (Fig. 3b: CL2120) and CL2659 (Fig. 3b: CL2659). At 100 µM, CQL reduced α-synuclein fluorescence in strain NL5901 (Fig. 3b: NL5901). At 100 µM and 50 µM CQL significantly prolonged the time until paralysis in strain CL2659 (Fig. 3b: CL2659). Neither 100 µM nor 10 µM CQL were able to prolong the lifespan in the wild type strain N2. Both concentrations even shortened it (Fig. 3b: N2). We studied the gene expression of metallothionein-1 (mt-1) and -2 (mt-2) after the CQL treatment in strain CL2120. The mt-1 and mt-2 expression remained unchanged in treated worms showing that the increased MT-2 protein level observed in CL2120 strain (Fig. 3b) must be a result of post-transcriptional regulation (Fig. 4a, b).

Emodin is a bioactive antraquinone present in some prescriptions of traditional Chinese medicine for cerebral protection activity. Cognitive deficits of hyperhomocysteinemia rats were improved by emodin. The animals had better behavioral performances, so that in the hippocampi the neuron loss decreased and synapse-related proteins increased. Further Aß overproduction and tau hyperphosphorylation were eliminated by emodin^52^. Emodin inhibited zinc-induced neurotoxicity in neuroblastoma SH-SY5Y cells^53^. Further Aloe-emodin has been shown to be neuroprotective to NMDA-treated retinal ganglion cells by Cu–Zn superoxide dismutase^54^. Anthraquinone-2-sulfonic acid prevents death of primary neurons by mechanisms like caspase inhibition and AKT activation. This compound may be a lead to develop a novel neurotherapeutic antraquinone-based drug^55^. In our study, emodin induced and prolonged MT release at 400 µM, 74 µM and 37 µM in CL2120 (Fig. 3c: CL2120) and CL2659 (Fig. 3c: CL2659-MT). α-synuclein expression was decreased with 37 µM emodin (Fig. 3c: NL5901) and when treated with 74 µM and 37 µM emodin the time until Aß expression-induced paralysis was prolonged in strain CL2659 (Fig. 3c: CL2659). At the highest dose (400 µM), emodin induced MT (Fig. 3c: CL2120), but was toxic in both CL2659 paralysis assay (Fig. 3c: CL2659) and NL5901 based Parkinson assay (Fig. 3c: NL5901). Bioactivity of 37 µM emodin in the paralysis assay has been lost when MT 2 has been knocked down with RNA interference and has been reduced when MT 1 has been knocked down (Fig. 3c: CL2659 with MT knockdown). Further 37 µM emodin prolonged lifespan in the wild type strain N2 (Fig. 3c: N2). We also studied the mt-1 and mt-2 expression in emodin treated CL2120 strain (Fig. 4a, b). We found a modest increase in mt-1 expression (Fig. 4a). The mt-1 expression showed an increasing trend in three and five day treated worms, while a reverse trend was observed for mt-2 (Fig. 4c). Our observations suggest that mt-1 and mt-2 may act differently to regulate the metallothionein levels in worms.

The test compound sesamin has been shown previously to act protective against Aß toxicity and to extend the lifespan in *C. elegans*^55^. We wanted to clarify if these effects were due to an increase of MT. Only a high dosage (300 µM) was able to induce MT expression (Fig. 3d: CL2120), but resulted in a significant toxicity in the PD assay (Fig. 3d: NL5901) and in the paralysis assay (Fig. 3d: CL2659). As reported before^55^ 56 µM and 28 µM sesamin were protective against Aß toxicity (Fig. 1d: CL2659) in our study, but α-synuclein fluorescence was unaltered (Fig. 3d: NL5901). Therefore, we assume that sesamin is protective through another mechanism than MT activation.

The paralysis assays performed with strain CL2659 showed that knockdown of either MT-1 or -2, alone, did not worsen the paralysis phenotype (Fig. 3c). Furthermore, quercetin (33 µM) treatment rescued the paralysis phenotype after MT-1 and MT-2 knockdown in CL2659 strain. Emodin (37 µM) mediated rescue was partly affected after MT-1 knockdown and was completely abolished after MT-2 knockdown in CL2659 strain. These observations indicate that MT-1 and MT-2 may show functional redundancy, however further studies are required to evaluate this hypothesis. Also, pharmacological compounds may act differently to ameliorate the paralysis symptoms, and precise mechanism of actions will be addressed in future work.

## Materials

### Equipment

Fluorescence microscope (Carl Zeiss, Austria), Pipetboy (LLG Labware by ISOLAB Laborgeräte GmbH, Germany), Flameboy (Integra, Germany), Bacterial Loop (VWR, Austria), Incubator (Lucky Reptile, Austria), Bigger Bill Digital Orbital Shaker—Barnstead Thermolyne M73735 (Clarkson laboratory & supply inc., USA), Freezer 4 °C (Liebherr ProfiLine, Germany), Freezer 16 °C (Öko-Santo Superelectronic, AEG, Austria), Vortexer (Janke & Kunkel IKA-Labortechnik, Germany), Jouan CR3-22 Centrifuge (ABI, France), Phase Contrast Microscope (Nikon TMS, Germany).

### Strains

All strains in this work were provided from CGC (Caenorhabditis Genetic Stock Centre), University of Minnesota, St. Paul, U.S.A. OP50: Uracil auxotroph Escherichia coli strain. CL2120: dvIs14 [unc-54/beta 1–42(pCL12) + mtl-2::gfp (pCL26)]. Mtl-2::gfp produces strong constitutive intestinal expression of GFP fused to metallothionein. This strain further expresses human Aß peptide. CL2122: dvIs15 [(pPD30.38) unc-54(vector) + (pCL26) mtl-2::gfp]. Control strain for CL2120. NL5901: pkIs2386 [unc54::α -synuclein::yfp unc-119(+). Expression of human a-synuclein fused to yellow fluorescent protein (YFP) in the body wall muscle of C. elegans, where it accumulates into “Lewy bodies” with increasing age. CL2659: dvIs770 [myo-3::Aß 1–42 wt::3′ UTR(long) + mtl-2::GFP]). N2: wild type strain. All substances were provided by Merck KGaA, Darmstadt, Germany.

*Escherichia colis* HT115(DE3) has been purchased from GE Dharmacon (www.horizondiscovery.com).

## Methods

### Maintenance of *C. elegans*

*Caenorhabditis elegans* were maintained according to the protocol of the CGC (Caenorhabditis Genetics Center), University of Minnesota, Minneapolis, MN 55455 USA. Cultivation and preparation of media and agar plates were done according the protocol of Stiernagle^56^. All worms are kept at 16 °C. *C. elegans* were growing on plates containing nematode growth medium (NGM) seeded with *Escherichia coli* strain OP50 (CGC) as described^57^. Large numbers of developmentally synchronized worms grew in solid culture and were harvested at L3 stage (CL2659) or L4 stage (CL2120, CL2122, NL5901). They were suspended at defined density in 96 well plates containing SOF medium (S-medium with OP50 and 5-fluorodeoxyuridine (FUdR)) and test compounds^58,59^. In vivo fluorescence was measured with a fluorescence microplate reader—phenotypical changes like paralysis with a stereo light microscope. In order to maintain an age-synchronized population we used the egg prep method from the CGC. To prevent the population from producing progeny, 5-fluorodeoxyuridine was used. In our procedure, a synchronized population is exposed to 60 µM FUdR just as it reaches sexual maturity^60^.

### Measuring the depletion of *E. coli* food source

OP50 were diluted 1:1 in liquid broth (LB) in a 96 well plate starting with a concentration of 6 mg/ml in triplicates and absorbance was measured at 600 nm (OD_600_)^34^ (Fig. 1d).

### Determination of optimal worm density and MT expression pattern

To follow protein expression in *C. elegans*, we used the transgenic strain CL2120, where MT is tagged to GFP and Aß is expressed. As control strain CL2122 was used. Real-time fluorescence intensity was measured with a fluorescence multiwell plate reader. For high throughput assays, the protocol of Leung et al.^59^ was used, where defined densities of developmentally synchronized fluorescent worms were added to 96-well plates (Fig. 1c). To determine the optimal worm concentration we serially diluted CL2120 L4 1:1 in a 96 well plate starting with a concentration of 256 worms per well and measured the fluorescence at 450/535 nm daily.

### MT assay with strain CL2120

50 µl SOF medium were added to each well of a 96 well plate. 10 µl of compounds dissolved in 1% DMSO were added in triplicates in different concentrations. L4 larvae were harvested from the NGM agar plates and suspended in SOF medium. 40 µl of worm suspension were added to each well of the 96 well plates at the concentration of 30–50 worms/well. MT expression was followed by measuring GFP at d0, d4, d8 by the fluorescent multiwell plate reader at 450/535 nm. OD_600_ was measured on d0 and d3 and d6 to observe depletion of food source. OP50 were added after 3 days at the time after the food source was usually depleted.

### Assaying α-synuclein toxicity with strain NL5901

The handling for the assays of alpha-synuclein toxicity followed the same protocol as used in the MT assay.

α-Synuclein expression was followed by measuring GFP at d0, d3 and d5 by the fluorescent multiwell plate reader at 450/535 nm. OD_600_ was measured on d0 and d3 to observe depletion of food source. OP50 were added after 3 days because this was the time after which food source was usually depleted.

### Assaying Aß toxicity CL2659: paralysis assay

In this strain Aβ expression can be induced by temperature upshift in muscle cells^59^. A correlation between the increase of neurotransmission and progression of paralysis has been reported previously^62^. This strain has wild-type movement at the permissive temperature of 16 °C but becomes paralysed upon temperature upshift to 25 °C within approximately 48 h in liquid culture. Treatments that inhibit Aβ toxicity in this model (e.g. exposure to *Ginkgo biloba* extracts^65^ alter the rate of paralysis in these worms. In accordance to the work of Dostal et al.^63^ with some alterations, we used the screening protocol for measuring the rate of paralysis. The inducible Aβ expression does not lead to amyloid deposits and the paralysis phenotype appears independent of amyloid deposition^64^. Therefore, the acute toxicity of induced Aβ expression resulting from the accumulation of soluble oligomeric Aβ can be measured. Simultaneously, real time MT expression was followed by GFP fluorescence. For a high throughput screening method 50 µl SO medium were added to each well of a 96 well plate. 10 µl of compounds dissolved in 1% DMSO were added in triplicates in different concentrations. L3 larvae were harvested from NGM plates and suspended in SO medium. 40 µl of worm suspension were added to each well of the 96 well plates in the concentration of 10–20 worms/well. Aβ transgene expression in muscle cells was induced by temperature upshift from 16 to 25 °C and lasts until the end of the paralysis assay. Usually on d0 (before temperature upshift) and d2 (48 h after temperature upshift) the number of paralysed worms was scored under the dissecting microscope. The percentage of non-paralysed worms on d0 and d2 is shown in a bar graph using MS-Excel 2010.

### MT induction in CL2659-MT

Fluorescence of GFP-tagged MT expressing worms in the paralysis assay was measured on d0 and d2 by the fluorescent multiwell plate reader at 450/535 nm and MS-Excel 2010 was used for all calculations and plotting of data. The changes in fluorescence between wells with worms treated with the test compound and vehicle control on two different days were analysed using two tailed Student’s t-test (n = 10–20/well).

### Lifespan assay with N2

The lifespan assays were performed with some alterations according the protocol of Solis and Petraschek^65^. L4 worms were washed from the agar plates, pelleted and transferred to a 96 well plate containing S-medium with OP50, FUdR and compounds in triplicates. Vehicle control contained 1% DMSO. Immediately number of worms were counted per well. Usually we used 10–20 worms/well. Counting after transferring worms to the 96-well plate marked time point 0. Counting of living worms was repeated after 18 days. After 6 days of transferring bacterial food source OP50 were added. After 18 days percentage of compound treated living worms were compared to worms from the vehicle control.

### MT 2 and MT 1 knockdown with RNA interference

Induction of RNA interference by feeding was performed according the protocol of Conte et al.^67^. Clones carried in E. coli HT115(DE3) were purchased from GE Dharmacon (www.horizondiscovery.com) . Clone Id for MT 1 is: K11G9.6 ORF and clone Id for MT 2 is T08G5.10 ORF. Synchronized L1 larvae of CL2659 were transferred to agar plates inoculated with the *E. coli* strain carrying the double stranded RNA for RNA interference. L3 larvae were harvested and paralysis assay was performed as usual.

### Gene expression analysis

Total RNA was extracted using TRIzol reagent^68^ (ThermoFisher scientific, Austria). Quantitative RT PCR was performed using the iTaq Universal SYBR Green Supermix (Bio-Rad Laboratories G.m.b.H., Austria) as per manufacturer’s instructions. Complementary DNA (cDNA) was synthesized with the ProtoScript II First Strand cDNA Synthesis Kit (New England Biolabs GmbH, Germany) using primers described by Chiang et al.^31^. Gene expression was standardized against rps-18 and comparative ΔΔCT method was used to measure the relative gene expression changes. All the experiments were performed in triplicates.

### Statistical evaluation of paralysis

Raw data of the paralysis assay were analysed in Excel to keep track of paralysed populations in each well. For each well the coordinates in the plate, strain, drug and the total number of animals paralysed on day 0 (d0) and day2 (d2) were recorded. For generation of bar graphs, the median fraction of non-paralysed worms was given as percentage at d0 and d2. Compound treated and vehicle treated worms were compared using two tailed Student’s-test (n = 10–20/well).

### Statistical evaluation of gene expression

For gene expression analysis, Graphpad Prism 8 was used to perform One-way ANOVA followed by Tukey's multiple comparisons test.

### Fluorescence analysis

The fluorescence intensity of each well was measured with a microplate reader with the appropriate emission and excitation wavelength (Filter for our assay: GFP 450/20ex 535/20ex). The difference of fluorescence increase or decrease between compound treated wells and vehicle control on two different days was analysed using two tailed Student’s t-test (n = 30–50/well).

## Data Availability

The datasets generated during and/or analysed during the current study are available from the corresponding author on reasonable request.
